# The Office Treatment of Hemorrhoids, Fistula, Etc.

**Published:** 1898-10

**Authors:** 


The Office Treatment of Hemorrhoids, Fistula, Etc., without Operation, Together with Remarks on the Relation of Diseases of the Rectum to Other Diseases in Both Sexes, but Especially in Women, and the Abuse of the Operation of Colostomy. By Charles B. Kelsey, A. M.,

M. D., late Professor of Surgery at the New York Post-Graduate Medical School and Hospital; Fellow of the New York Academy of Medicine, the New York County Medical Society, etc. E. R. Pelton, Publisher, No. 19 East Sixteenth Street, New York, 1898.

This little volume of sixty-eight pages is what its title above copied shows it to be, an argument for the treatment of hemorrhoids and fistula without operation. In this argument are also included operations for the cure of obstruction of the bowel without resorting to artificial anus. The book is, to our thinking, unsatisfactory, because it gives no details of the treatment, being limited only to the argument. An author of the eminence in his profession of Dr. Kelsey could make a much better book than the one we now have before us, by elaborating the actual steps he takes to achieve the results he claims.

